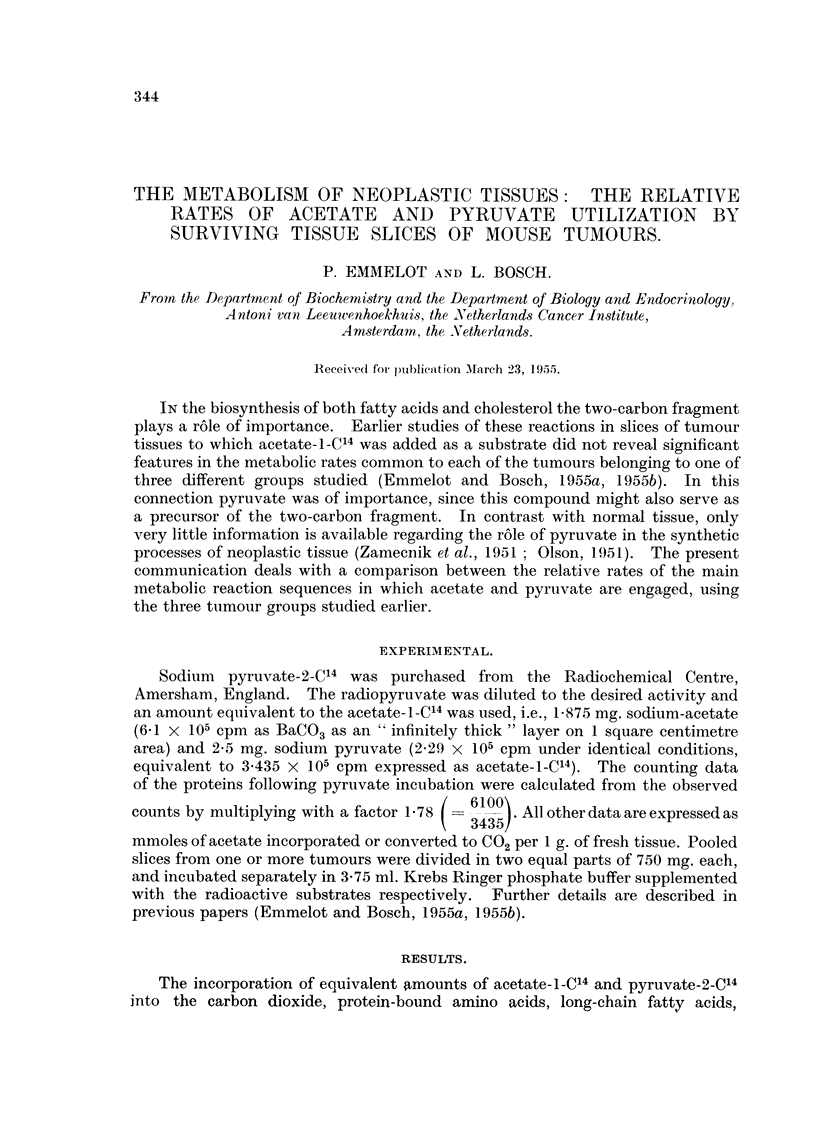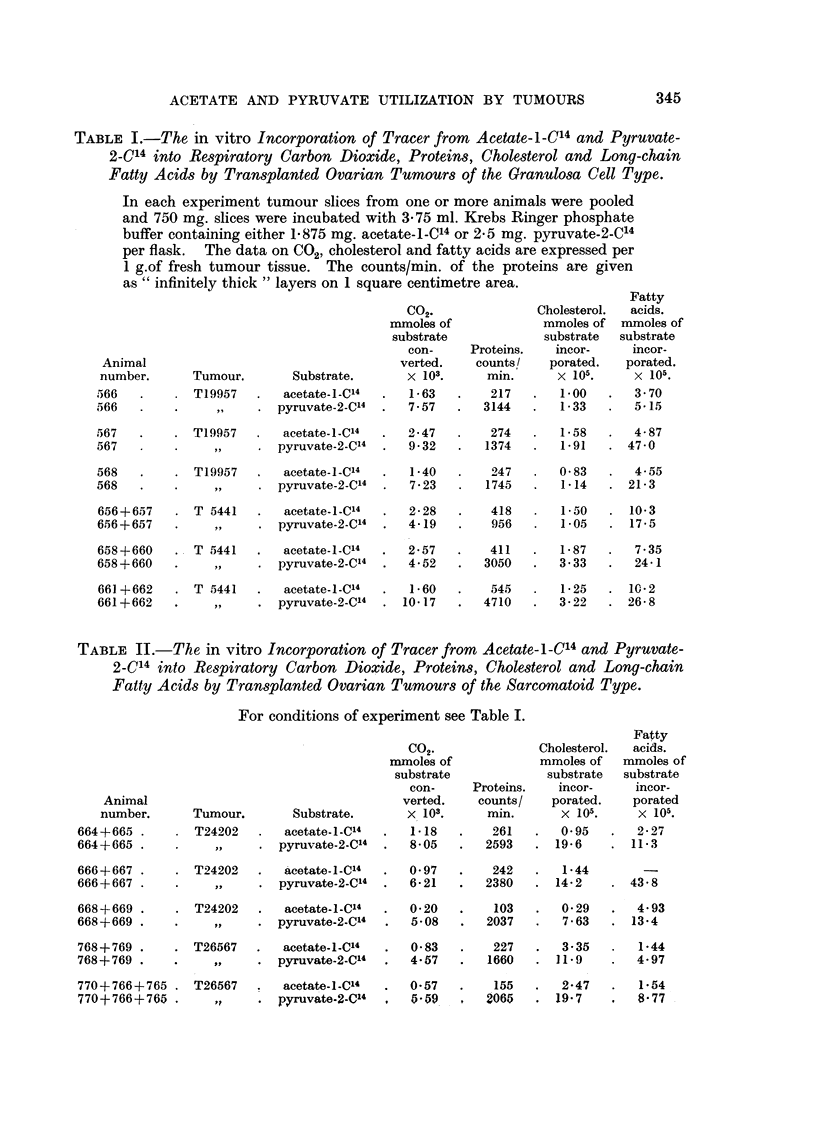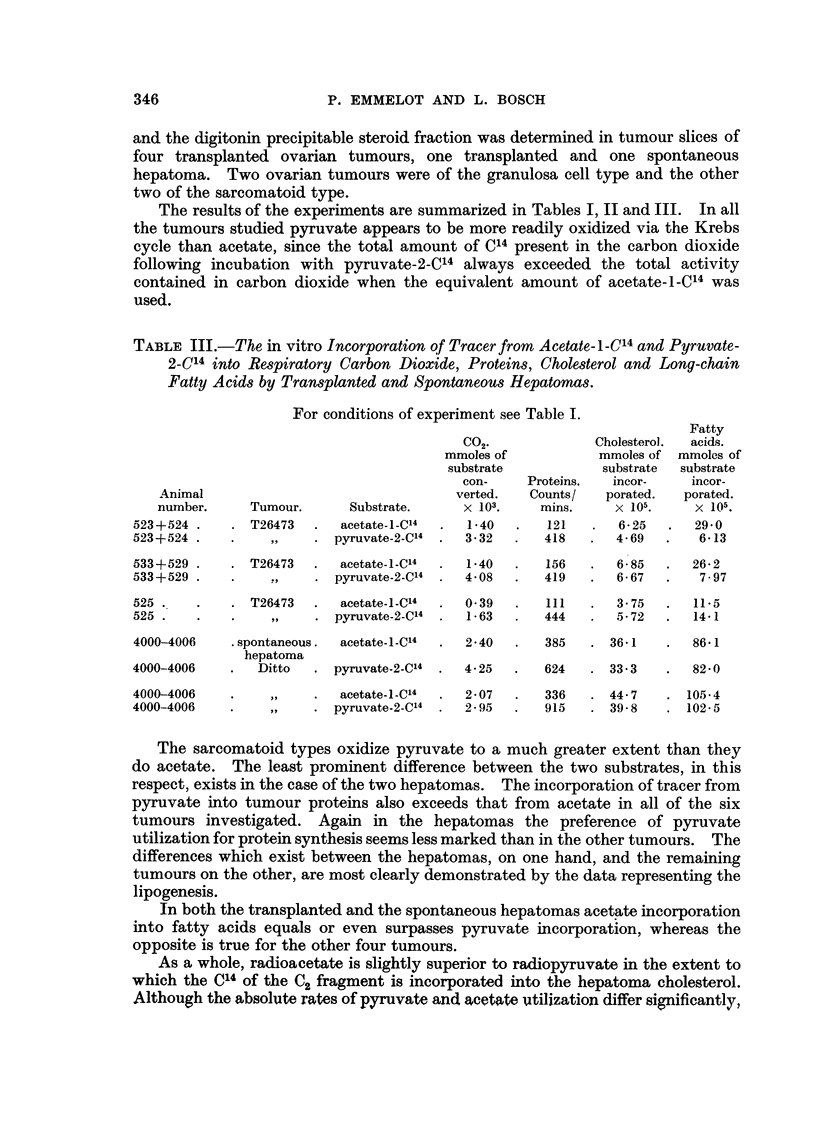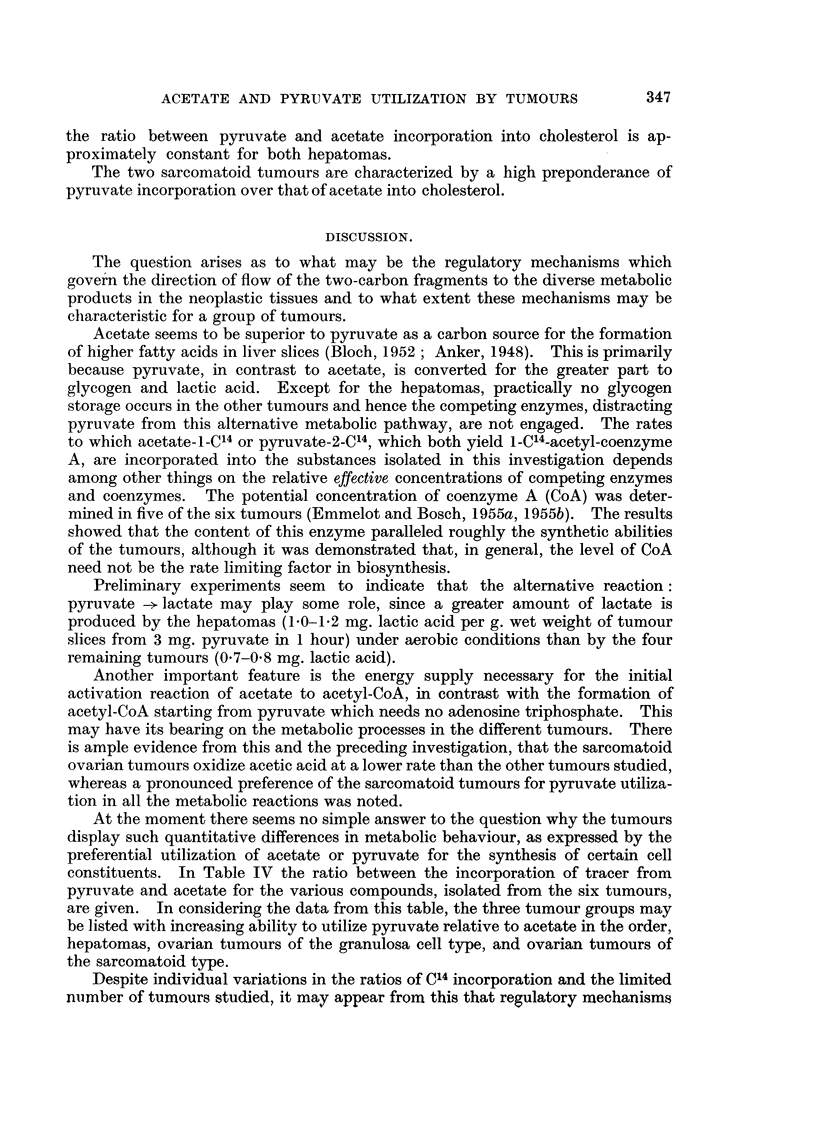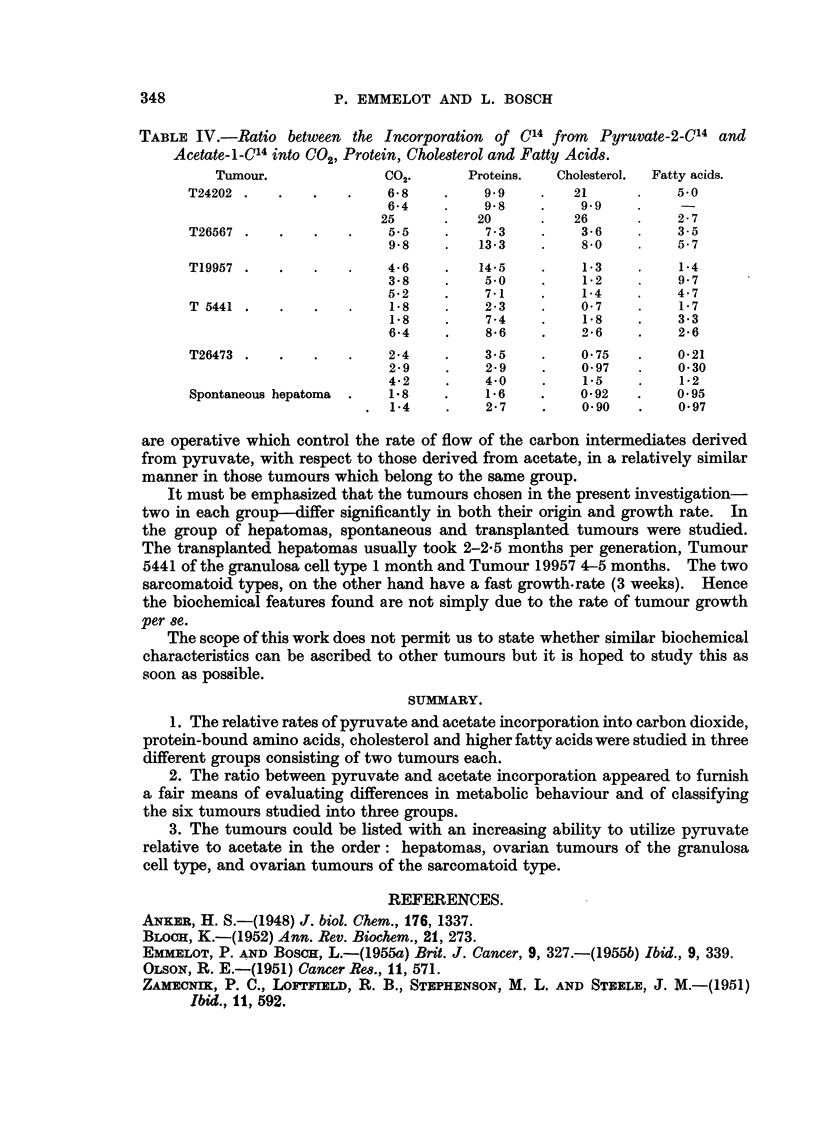# The Metabolism of Neoplastic Tissues: The Relative Rates of Acetate and Pyruvate Utilization by Surviving Tissue Slices of Mouse Tumours

**DOI:** 10.1038/bjc.1955.32

**Published:** 1955-06

**Authors:** P. Emmelot, L. Bosch


### THE METABOLISM OF NEOPLASTIC TISSUES: THE RELATIVE RATES OF ACETATE AND PYRUVATE UTILIZATION BY SURVIVING TISSUE SLICES OF MOUSE TUMOURS.

P. EMMELOT AND L. BOSCH.

*From the Department of Biochemistry and the Department of Biology and Endocrinology, Antoni van Leeuwenhoekhuis, the Netherlands Cancer Institute, Amsterdam, the Netherlands.*

Received for publication March 23, 1955.

IN the biosynthesis of both fatty acids and cholesterol the two-carbon fragment plays a rôle of importance. Earlier studies of these reactions in slices of tumour tissues to which acetate-1-$C^{14}$ was added as a substrate did not reveal significant features in the metabolic rates common to each of the tumours belonging to one of three different groups studied (Emmelot and Bosch, 1955*a*, 1955*b*). In this connection pyruvate was of importance, since this compound might also serve as a precursor of the two-carbon fragment. In contrast with normal tissue, only very little information is available regarding the rôle of pyruvate in the synthetic processes of neoplastic tissue (Zamecnik *et al.*, 1951 ; Olson, 1951). The present communication deals with a comparison between the relative rates of the main metabolic reaction sequences in which acetate and pyruvate are engaged, using the three tumour groups studied earlier.

### EXPERIMENTAL.

Sodium pyruvate-2-$C^{14}$ was purchased from the Radiochemical Centre, Amersham, England. The radiopyruvate was diluted to the desired activity and an amount equivalent to the acetate-1-$C^{14}$ was used, i.e., 1·875 mg. sodium-acetate ($6{\cdot}1 \times 10^5$ cpm as $BaCO_3$ as an " infinitely thick " layer on 1 square centimetre area) and 2·5 mg. sodium pyruvate ($2{\cdot}29 \times 10^5$ cpm under identical conditions, equivalent to $3{\cdot}435 \times 10^5$ cpm expressed as acetate-1-$C^{14}$). The counting data of the proteins following pyruvate incubation were calculated from the observed counts by multiplying with a factor 1·78 $\left(= \frac{6100}{3435}\right)$. All other data are expressed as mmoles of acetate incorporated or converted to $CO_2$ per 1 g. of fresh tissue. Pooled slices from one or more tumours were divided in two equal parts of 750 mg. each, and incubated separately in 3·75 ml. Krebs Ringer phosphate buffer supplemented with the radioactive substrates respectively. Further details are described in previous papers (Emmelot and Bosch, 1955*a*, 1955*b*).

### RESULTS.

The incorporation of equivalent amounts of acetate-1-$C^{14}$ and pyruvate-2-$C^{14}$ into the carbon dioxide, protein-bound amino acids, long-chain fatty acids,

TABLE I.—*The* in vitro *Incorporation of Tracer from Acetate*-1-$C^{14}$ *and Pyruvate*-2-$C^{14}$ *into Respiratory Carbon Dioxide, Proteins, Cholesterol and Long-chain Fatty Acids by Transplanted Ovarian Tumours of the Granulosa Cell Type.*

In each experiment tumour slices from one or more animals were pooled and 750 mg. slices were incubated with 3·75 ml. Krebs Ringer phosphate buffer containing either 1·875 mg. acetate-1-$C^{14}$ or 2·5 mg. pyruvate-2-$C^{14}$ per flask. The data on $CO_2$, cholesterol and fatty acids are expressed per 1 g.of fresh tumour tissue. The counts/min. of the proteins are given as " infinitely thick " layers on 1 square centimetre area.

| Animal number. | Tumour. | Substrate. | $CO_2$. mmoles of substrate converted. $\times 10^3$. | Proteins. counts/min. | Cholesterol. mmoles of substrate incorporated. $\times 10^5$. | Fatty acids. mmoles of substrate incorporated. $\times 10^5$. |
|---|---|---|---|---|---|---|
| 566 | T19957 | acetate-1-$C^{14}$ | 1·63 | 217 | 1·00 | 3·70 |
| 566 | ,, | pyruvate-2-$C^{14}$ | 7·57 | 3144 | 1·33 | 5·15 |
| 567 | T19957 | acetate-1-$C^{14}$ | 2·47 | 274 | 1·58 | 4·87 |
| 567 | ,, | pyruvate-2-$C^{14}$ | 9·32 | 1374 | 1·91 | 47·0 |
| 568 | T19957 | acetate-1-$C^{14}$ | 1·40 | 247 | 0·83 | 4·55 |
| 568 | ,, | pyruvate-2-$C^{14}$ | 7·23 | 1745 | 1·14 | 21·3 |
| 656+657 | T 5441 | acetate-1-$C^{14}$ | 2·28 | 418 | 1·50 | 10·3 |
| 656+657 | ,, | pyruvate-2-$C^{14}$ | 4·19 | 956 | 1·05 | 17·5 |
| 658+660 | T 5441 | acetate-1-$C^{14}$ | 2·57 | 411 | 1·87 | 7·35 |
| 658+660 | ,, | pyruvate-2-$C^{14}$ | 4·52 | 3050 | 3·33 | 24·1 |
| 661+662 | T 5441 | acetate-1-$C^{14}$ | 1·60 | 545 | 1·25 | 10·2 |
| 661+662 | ,, | pyruvate-2-$C^{14}$ | 10·17 | 4710 | 3·22 | 26·8 |

TABLE II.—*The* in vitro *Incorporation of Tracer from Acetate*-1-$C^{14}$ *and Pyruvate*-2-$C^{14}$ *into Respiratory Carbon Dioxide, Proteins, Cholesterol and Long-chain Fatty Acids by Transplanted Ovarian Tumours of the Sarcomatoid Type.*

For conditions of experiment see Table I.

| Animal number. | Tumour. | Substrate. | $CO_2$. mmoles of substrate converted. $\times 10^3$. | Proteins. counts/min. | Cholesterol. mmoles of substrate incorporated. $\times 10^5$. | Fatty acids. mmoles of substrate incorporated $\times 10^5$. |
|---|---|---|---|---|---|---|
| 664+665 | T24202 | acetate-1-$C^{14}$ | 1·18 | 261 | 0·95 | 2·27 |
| 664+665 | ,, | pyruvate-2-$C^{14}$ | 8·05 | 2593 | 19·6 | 11·3 |
| 666+667 | T24202 | acetate-1-$C^{14}$ | 0·97 | 242 | 1·44 | — |
| 666+667 | ,, | pyruvate-2-$C^{14}$ | 6·21 | 2380 | 14·2 | 43·8 |
| 668+669 | T24202 | acetate-1-$C^{14}$ | 0·20 | 103 | 0·29 | 4·93 |
| 668+669 | ,, | pyruvate-2-$C^{14}$ | 5·08 | 2037 | 7·63 | 13·4 |
| 768+769 | T26567 | acetate-1-$C^{14}$ | 0·83 | 227 | 3·35 | 1·44 |
| 768+769 | ,, | pyruvate-2-$C^{14}$ | 4·57 | 1660 | 11·9 | 4·97 |
| 770+766+765 | T26567 | acetate-1-$C^{14}$ | 0·57 | 155 | 2·47 | 1·54 |
| 770+766+765 | ,, | pyruvate-2-$C^{14}$ | 5·59 | 2065 | 19·7 | 8·77 |

and the digitonin precipitable steroid fraction was determined in tumour slices of four transplanted ovarian tumours, one transplanted and one spontaneous hepatoma. Two ovarian tumours were of the granulosa cell type and the other two of the sarcomatoid type.

The results of the experiments are summarized in Tables I, II and III. In all the tumours studied pyruvate appears to be more readily oxidized via the Krebs cycle than acetate, since the total amount of $C^{14}$ present in the carbon dioxide following incubation with pyruvate-2-$C^{14}$ always exceeded the total activity contained in carbon dioxide when the equivalent amount of acetate-1-$C^{14}$ was used.

TABLE III.—*The* in vitro *Incorporation of Tracer from Acetate-1-$C^{14}$ and Pyruvate-2-$C^{14}$ into Respiratory Carbon Dioxide, Proteins, Cholesterol and Long-chain Fatty Acids by Transplanted and Spontaneous Hepatomas.*

For conditions of experiment see Table I.

| Animal number. | Tumour. | Substrate. | $CO_2$. mmoles of substrate converted. $\times 10^3$. | Proteins. Counts/mins. | Cholesterol. mmoles of substrate incorporated. $\times 10^5$. | Fatty acids. mmoles of substrate incorporated. $\times 10^5$. |
|---|---|---|---|---|---|---|
| 523+524 | T26473 | acetate-1-$C^{14}$ | 1·40 | 121 | 6·25 | 29·0 |
| 523+524 | ,, | pyruvate-2-$C^{14}$ | 3·32 | 418 | 4·69 | 6·13 |
| 533+529 | T26473 | acetate-1-$C^{14}$ | 1·40 | 156 | 6·85 | 26·2 |
| 533+529 | ,, | pyruvate-2-$C^{14}$ | 4·08 | 419 | 6·67 | 7·97 |
| 525 | T26473 | acetate-1-$C^{14}$ | 0·39 | 111 | 3·75 | 11·5 |
| 525 | ,, | pyruvate-2-$C^{14}$ | 1·63 | 444 | 5·72 | 14·1 |
| 4000–4006 | spontaneous hepatoma | acetate-1-$C^{14}$ | 2·40 | 385 | 36·1 | 86·1 |
| 4000–4006 | Ditto | pyruvate-2-$C^{14}$ | 4·25 | 624 | 33·3 | 82·0 |
| 4000–4006 | ,, | acetate-1-$C^{14}$ | 2·07 | 336 | 44·7 | 105·4 |
| 4000–4006 | ,, | pyruvate-2-$C^{14}$ | 2·95 | 915 | 39·8 | 102·5 |

The sarcomatoid types oxidize pyruvate to a much greater extent than they do acetate. The least prominent difference between the two substrates, in this respect, exists in the case of the two hepatomas. The incorporation of tracer from pyruvate into tumour proteins also exceeds that from acetate in all of the six tumours investigated. Again in the hepatomas the preference of pyruvate utilization for protein synthesis seems less marked than in the other tumours. The differences which exist between the hepatomas, on one hand, and the remaining tumours on the other, are most clearly demonstrated by the data representing the lipogenesis.

In both the transplanted and the spontaneous hepatomas acetate incorporation into fatty acids equals or even surpasses pyruvate incorporation, whereas the opposite is true for the other four tumours.

As a whole, radioacetate is slightly superior to radiopyruvate in the extent to which the $C^{14}$ of the $C_2$ fragment is incorporated into the hepatoma cholesterol. Although the absolute rates of pyruvate and acetate utilization differ significantly, the ratio between pyruvate and acetate incorporation into cholesterol is approximately constant for both hepatomas.

The two sarcomatoid tumours are characterized by a high preponderance of pyruvate incorporation over that of acetate into cholesterol.

### DISCUSSION.

The question arises as to what may be the regulatory mechanisms which govern the direction of flow of the two-carbon fragments to the diverse metabolic products in the neoplastic tissues and to what extent these mechanisms may be characteristic for a group of tumours.

Acetate seems to be superior to pyruvate as a carbon source for the formation of higher fatty acids in liver slices (Bloch, 1952 ; Anker, 1948). This is primarily because pyruvate, in contrast to acetate, is converted for the greater part to glycogen and lactic acid. Except for the hepatomas, practically no glycogen storage occurs in the other tumours and hence the competing enzymes, distracting pyruvate from this alternative metabolic pathway, are not engaged. The rates to which acetate-1-$C^{14}$ or pyruvate-2-$C^{14}$, which both yield 1-$C^{14}$-acetyl-coenzyme A, are incorporated into the substances isolated in this investigation depends among other things on the relative *effective* concentrations of competing enzymes and coenzymes. The potential concentration of coenzyme A (CoA) was determined in five of the six tumours (Emmelot and Bosch, 1955*a*, 1955*b*). The results showed that the content of this enzyme paralleled roughly the synthetic abilities of the tumours, although it was demonstrated that, in general, the level of CoA need not be the rate limiting factor in biosynthesis.

Preliminary experiments seem to indicate that the alternative reaction : pyruvate $\rightarrow$ lactate may play some role, since a greater amount of lactate is produced by the hepatomas (1·0–1·2 mg. lactic acid per g. wet weight of tumour slices from 3 mg. pyruvate in 1 hour) under aerobic conditions than by the four remaining tumours (0·7–0·8 mg. lactic acid).

Another important feature is the energy supply necessary for the initial activation reaction of acetate to acetyl-CoA, in contrast with the formation of acetyl-CoA starting from pyruvate which needs no adenosine triphosphate. This may have its bearing on the metabolic processes in the different tumours. There is ample evidence from this and the preceding investigation, that the sarcomatoid ovarian tumours oxidize acetic acid at a lower rate than the other tumours studied, whereas a pronounced preference of the sarcomatoid tumours for pyruvate utilization in all the metabolic reactions was noted.

At the moment there seems no simple answer to the question why the tumours display such quantitative differences in metabolic behaviour, as expressed by the preferential utilization of acetate or pyruvate for the synthesis of certain cell constituents. In Table IV the ratio between the incorporation of tracer from pyruvate and acetate for the various compounds, isolated from the six tumours, are given. In considering the data from this table, the three tumour groups may be listed with increasing ability to utilize pyruvate relative to acetate in the order, hepatomas, ovarian tumours of the granulosa cell type, and ovarian tumours of the sarcomatoid type.

Despite individual variations in the ratios of $C^{14}$ incorporation and the limited number of tumours studied, it may appear from this that regulatory mechanisms

TABLE IV.—*Ratio between the Incorporation of $C^{14}$ from Pyruvate-2-$C^{14}$ and Acetate-1-$C^{14}$ into $CO_2$, Protein, Cholesterol and Fatty Acids.*

| Tumour. | $CO_2$. | Proteins. | Cholesterol. | Fatty acids. |
|---|---|---|---|---|
| T24202 | 6·8 | 9·9 | 21 | 5·0 |
| | 6·4 | 9·8 | 9·9 | — |
| | 25 | 20 | 26 | 2·7 |
| T26567 | 5·5 | 7·3 | 3·6 | 3·5 |
| | 9·8 | 13·3 | 8·0 | 5·7 |
| T19957 | 4·6 | 14·5 | 1·3 | 1·4 |
| | 3·8 | 5·0 | 1·2 | 9·7 |
| | 5·2 | 7·1 | 1·4 | 4·7 |
| T 5441 | 1·8 | 2·3 | 0·7 | 1·7 |
| | 1·8 | 7·4 | 1·8 | 3·3 |
| | 6·4 | 8·6 | 2·6 | 2·6 |
| T26473 | 2·4 | 3·5 | 0·75 | 0·21 |
| | 2·9 | 2·9 | 0·97 | 0·30 |
| | 4·2 | 4·0 | 1·5 | 1·2 |
| Spontaneous hepatoma | 1·8 | 1·6 | 0·92 | 0·95 |
| | 1·4 | 2·7 | 0·90 | 0·97 |

are operative which control the rate of flow of the carbon intermediates derived from pyruvate, with respect to those derived from acetate, in a relatively similar manner in those tumours which belong to the same group.

It must be emphasized that the tumours chosen in the present investigation—two in each group—differ significantly in both their origin and growth rate. In the group of hepatomas, spontaneous and transplanted tumours were studied. The transplanted hepatomas usually took 2–2·5 months per generation, Tumour 5441 of the granulosa cell type 1 month and Tumour 19957 4–5 months. The two sarcomatoid types, on the other hand have a fast growth-rate (3 weeks). Hence the biochemical features found are not simply due to the rate of tumour growth *per se*.

The scope of this work does not permit us to state whether similar biochemical characteristics can be ascribed to other tumours but it is hoped to study this as soon as possible.

### SUMMARY.

1. The relative rates of pyruvate and acetate incorporation into carbon dioxide, protein-bound amino acids, cholesterol and higher fatty acids were studied in three different groups consisting of two tumours each.

2. The ratio between pyruvate and acetate incorporation appeared to furnish a fair means of evaluating differences in metabolic behaviour and of classifying the six tumours studied into three groups.

3. The tumours could be listed with an increasing ability to utilize pyruvate relative to acetate in the order : hepatomas, ovarian tumours of the granulosa cell type, and ovarian tumours of the sarcomatoid type.

### REFERENCES.

ANKER, H. S.—(1948) *J. biol. Chem.*, **176**, 1337.
BLOCH, K.—(1952) *Ann. Rev. Biochem.*, **21**, 273.
EMMELOT, P. AND BOSCH, L.—(1955*a*) *Brit. J. Cancer*, **9**, 327.—(1955*b*) *Ibid.*, **9**, 339.
OLSON, R. E.—(1951) *Cancer Res.*, **11**, 571.
ZAMECNIK, P. C., LOFTFIELD, R. B., STEPHENSON, M. L. AND STEELE, J. M.—(1951) *Ibid.*, **11**, 592.